# ^18^F-FLT PET/CT imaging in a Wister rabbit inflammation model

**DOI:** 10.3892/etm.2014.1687

**Published:** 2014-04-24

**Authors:** YEYING TAN, JUN LIANG, DEFENG LIU, FENG ZHU, GUANMIN WANG, XUEMEI DING, CONGHUI HAN

**Affiliations:** 1 PET-CT Center, The Affiliated School of Clinical Medicine of Xuzhou Medical College, Xuzhou, Jiangsu 221009, P.R. China; 2 Department of Urology, The Affiliated School of Clinical Medicine of Xuzhou Medical College, Xuzhou, Jiangsu 221009, P.R. China

**Keywords:** 3′-deoxy-3′-^18^F-fluorothymidine, *Staphylococcus aureus* model, gentamicin + calcium sulphate model

## Abstract

The aim of the present study was to determine the tumour specificity of the newly developed nucleoside metabolic positron emission tomography (PET) tracer, 3′-deoxy-3′-^18^F-fluorothymidine (^18^F-FLT). Using ^18^F-FLT PET imaging, DNA synthesis and cell proliferation were detected in *Staphylococcus aureus (S. aureus)* abscess and calcium sulphate models in Wister rabbits. A total of eight rabbits were implanted with *S. aureus* in the left tibia to induce an inflammatory process. Calcium sulphate + gentamicin was implanted in the right tibia to induce a physical stimulus without bacterial multiplication. After four weeks, the animals underwent ^18^F-FLT PET imaging, bacterial culturing and tissue pathology. The uptake of ^18^F-FLT was significantly higher in the abscess site compared with that in the granuloma, with maximum standardised uptake values of 5.76±0.25 and 1.15±0.32, respectively (P<0.01). This indicates that ^18^F-FLT is not a specific tumour tracer since active inflammation also results in the uptake of this compound. However, the tumour specificity of this tracer is higher compared with that of ^18^F-fluorodeoxyglucose. Therefore, ^18^F-FLT may be useful in the differential diagnosis of benign and malignant tumours.

## Introduction

The differential diagnosis of benign and malignant tumours is challenging in clinical practice. Positron emission tomography (PET) and computed tomography (CT) are revolutionary in the field of medicine since these procedures are able to detect the metabolism of glucose, amino acids and macromolecules, enabling the diagnosis of diseases at the molecular and genetic levels (1,2).

^18^F-fluorodeoxyglucose (^18^F-FDG) is the most widely used tracer. During glycolysis, the expression levels of the glucose transporter protein increase, resulting in the ^18^F-FDG uptake by malignant tumours being higher compared with that of normal tissues (3,4). However, ^18^F-FDG is not a tumour-specific tracer and may produce certain false-positive results (5,6).

In previous years, numerous studies have focused on the research and development of nucleoside metabolic tracers, of which 3′-deoxy-3′-^18^F-fluorothymidine (^18^F-FLT) is the most promising. FLT is a pyrimidine analogue that is catalysed by thymidine kinase 1 (TK1) to induce the phosphorylation of FLT into a single phosphate and an intracellular strand. TK1 is a key enzyme in the salvage synthesis pathway of DNA and TK1 activity in malignant tumour cells is 3–10-fold higher than in benign cells (7–9). Therefore, TK1 activity indirectly reflects the state of tumour cell proliferation (10).

The majority of studies consider ^18^F-FLT to have 100% specificity for malignant tumours (11). However, cell proliferation is not tumour-specific and bacterial and viral growth also involve DNA replication. In addition, a previous study reported a case of ^18^F-FLT uptake in pulmonary tuberculosis (12,13). However, the animal models used in previous studies have certain limitations (12,13). In one study (12), inflammation was induced by turpentine, which is a physical or chemical stimulus and causes nonbacterial inflammation. In the other study (13), the experiment did not include a bacterial breeding period. Therefore, the design of a variety of models of inflammation is required, with ^18^F-FLT imaging being performed at various time points to determine the specificity of ^18^F-FLT for tumour cells.

## Materials and methods

### Experimental animals

Rabbits (age, 6–8 weeks; T739 inbred strain; either gender; weight, 22–25 g) were provided by the Cancer Institute of the Chinese Academy of Medical Science (SCXK11-00-0005; Beijing, China). The study was conducted in strict accordance with the recommendations in the Guide for the Care and Use of Laboratory Animals of the National Institutes of Health (1995). The animal use protocol was reviewed and approved by the Institutional Animal Care and Use Committee of Xuzhou Central Hospital (Xuzhou, China).

### Experimental design

The experimental animals were divided into groups A and B, with eight rabbits in each group. Group A (right tibia) was implanted with calcium sulphate + gentamicin tablets, whereas group B (left tibia) was implanted with *Staphylococcus aureus (S. aureus)*.

### Establishment of an inflammation model

Animals were anaesthetised with a mixture of 2 ml ketamine and 1.5 ml sumianxin (MeiDa high-tech company, Beijing, China) at 0.3 ml/kg body weight. For local skin preparation, the animals were fixed in a supine position, regularly disinfected and then covered with sterile towels. Next, a tibial patellar medial inferior longitudinal incision, ~3 cm in length, was made to expose the medial proximal tibia, and a window was created in the proximal tibia using a 1-mm diameter drill bit. Intramedullary tissue and a section of the cancellous bone were scraped with a small curette to create a 3×10 mm rectangular bone window. Based on the experimental design, group A received gentamicin sustained-release tablets (2 mg; MeiDa high-tech company) and calcium sulphate (2 mg), whereas group B was injected with 0.15 ml sodium salts of the fatty acids of cod liver oil (MeiDa high-tech company), 0.2 ml *S. aureus* (5×10^6^ CFU) and 1 ml physiological saline. The bone window was then closed with bone wax and the wounds were sutured after flushing with physiological saline. Following surgery, the animals were fed a conventional diet for four weeks. No animal was administered intravenous or oral antibiotic therapy.

### PET imaging

^18^F-FLT PET (Phillips, Amsterdam, Netherlands) imaging was performed four weeks following surgery. The animals were injected with 50 μCi/100 μl ^18^F-FLT in the tail vein and were subjected to general anaesthesia via the intramuscular injection of a mixed solution of 2 ml ketamine and 1.5 ml sumianxin. After 10 min, the animals were fixed in an ECAT Exact HR+ PET imaging system (Siemens AG, Munich, Germany) that was equipped with a 3D mode image acquisition system. Following dispersion, random counting and dead time correction, all measurements were analysed through the reconstruction of coronal, transverse and sagittal sectional images by the filter-rejected projection method.

### Gross observation

Following anaesthesia, the animals underwent surgery and samples were collected. No inflammation of the local limb soft tissue, bone tissue or local pus was observed. Tibia samples of ~2 cm were collected to identify abnormal changes in the marrow cavity. The samples were divided into two sections to monitor the development of bone defects and osteomyelitis.

### Histological observation

Specimens were fixed with 10% neutral formaldehyde and decalcified for 20 days using a decalcifying fluid containing EDTA. The samples were then embedded in paraffin, conventionally sectioned and subsequently stained with hematoxylin and eosin. A light microscope was used to observe the repair of bone defects, as well as bone infection, local abscess, inflammatory cell infiltration, dead bone and new bone formation.

### Bacterial and cancer cell culture

Reproductive-stage *Escherichia coli (E. coli)* and A549 lung cancer cells were incubated with ^18^F-FLT for 1 h in RMPI 1640 conataining 13% IV-type collagenase and 10% FBS (Gibco, Carlsbad, CA, USA). The samples were then centrifuged and the supernatant was removed. A γ-well counter (162 factory, Beijing, China) was then used to determine the radioactive counts.

### Statistical analysis

Statistical analyses were performed using the SPSS statistical software, version 12.0 (SPSS, Inc., Chicago, IL, USA). Data between the FLT and calcium sulphate groups were compared using a paired t-test. P<0.05 was considered to indicate a statistically significant difference.

## Results

### General observations

In group A, no bone destruction or marrow cavity pus was identified in the medullary cavity. The animals in this group exhibited normal bone tissue, red colouration, loose tissue, clear boundaries of the intramedullary tissue and cortical bone, wreckage remains of the implanted calcium sulphate following partial degradation, largely degraded antibiotic sustained-release tablets and mostly repaired defects.

In group B, local soft tissue inflammatory oedema was observed and the control group exhibited intramedullary tissue swelling, a large amount of pus, necrotic intramedullary tissue and dead bone formation in the proximal tibial specimen. A large number of *S. aureus* bacteria were visible in the bacterial culture.

### Histological examination

Bone tissue sections were demineralised four weeks following surgery and histological changes in the bone pathology were observed under a light microscope.

**Figure 1:**
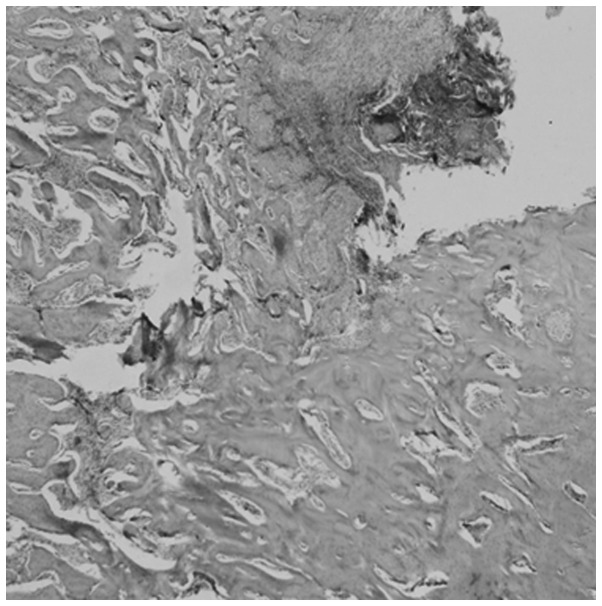
Calcium sulphate and degradation products are surrounded by fibrous tissue. No inflammatory cells, bone necrosis or local abscesses are visible in the medullary cavity. Haematoxylin and eosin staining. Magnification, ×4.

**Figure 2:**
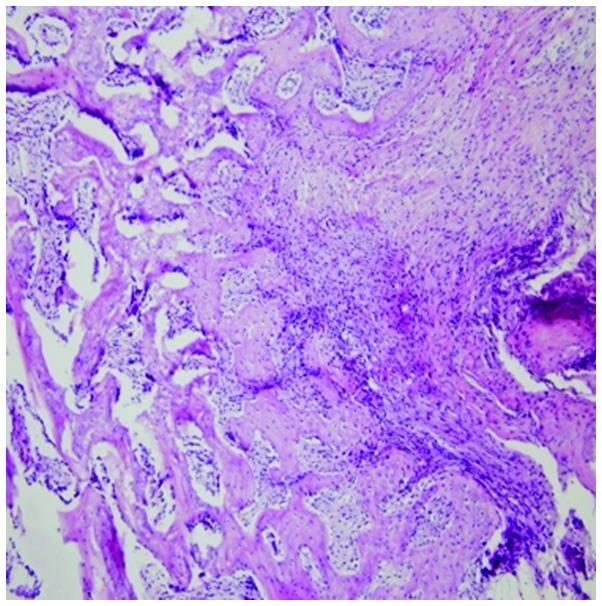
Inflammatory cells and *Staphylococcus aureus* abscess formation are visible, as well as a small amount of new bone formation, primarily replacing the scar tissue. Haematoxylin and eosin staining. Magnification, ?

In group A, calcium sulphate and the degradation products were surrounded by fibrous tissue and there were no inflammatory cells, necrosis of the bone or local abscesses present in the medullary cavity (Fig. 1). In group B, a certain amount of inflammatory cells and *S. aureus* abscess formation were visible. In addition, a small degree of new bone formation over the scar tissues was observed (Fig. 2).

### PET imaging

**Figure 3:**
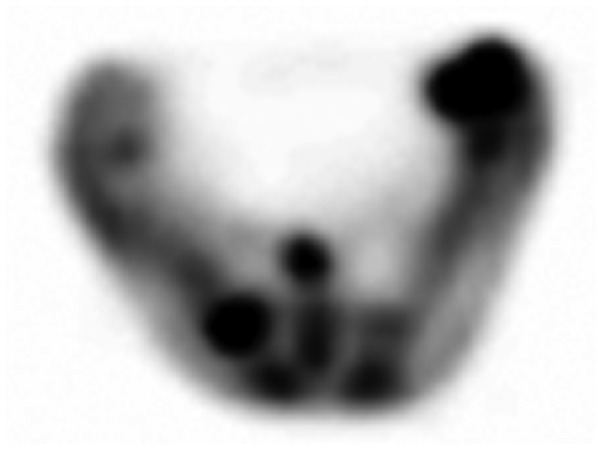
^18^F-FLT inflammation model imaging (transverse). The uptake of ^18^F-FLT in the abscess was markedly higher compared with that in the granulomatous tissue. ^18^F-FLT, 3′-deoxy-3′-18F-fluorothymidine.

Uptake of ^18^F-FLT by the abscess was markedly higher compared with that by the granuloma [maximum standardised uptake value (SUV_max_), 5.76±0.25 and 1.15±0.32, respectively; P<0.01; Fig. 3].

### Bacterial and cancer cell culture

The ^18^F-FLT uptake by E. *coli* was five times higher compared with that by tumour cells (35,680 and 7,200, respectively; P<0.01).

## Discussion

^18^F-FDG, which has been referred to as the ‘molecule of the century,’ is a highly sensitive tracer, but is prone to producing false-positive results (14). ^18^F-FLT is an analogue of thymidine, which is involved in DNA synthesis and is an indicator of cell proliferation (15). The majority of studies consider the tumour specificity of this tracer to be 100% (16–18). Carter *et al* (11) used ^18^F-FLT and ^18^F-FDG to study inflammation and tumour models in rats. The rat inflammation model was prepared through the injection of *Escherichia coli (E. coli)* in the rat thigh, while the tumour model was thigh sarcoma. The results indicated that the ^18^F-FDG tumour/non-tumour ratio was 7.39, which was higher than the 2.76 of ^18^F-FLT. ^18^F-FDG uptake was slightly lower in inflammatory tissues compared with that in tumour tissues; the inflammation-to-normal tissue ratio was 3.29, whereas the ^18^F-FLT inflammatory tissue/normal tissue uptake ratio was only 1.14. van Waarde *et al* (12) also compared ^18^F-FLT and ^18^F-FDG uptake in inflammatory and tumour tissues in a rat model. The tumour model was prepared by injecting C6 nerve glioma into the right shoulder of the rats, while the inflammation model was established by injecting turpentine oil into the left calf. In this study (12), the tumour/muscle ratio of ^18^F-FDG was 13, which was higher than the 8 of ^18^F-FLT. The radioactive uptake of ^18^F-FDG significantly increased at the site of inflammation, whereas no significant uptake of ^18^F-FLT was observed in the inflammatory sites.

It may be hypothesised that the *E. coli* implanted in the study by Carter *et al* (11) was undetectable at the reproductive stage. Turpentine is an aseptic chemical irritant that causes local plasma extravasation and neutrophil migration. These inflammatory cells originate in the blood. The site of inflammation contains no bacteria and, thus, does not accurately reflect bacterial inflammation.

In the present study, bacteria was shown to take up a large amount of ^18^F-FLT in the reproductive stage, as shown by the *E. coli* cell culture assay and the rabbit *S. aureus* abscess model. These results indicate that ^18^F-FLT is not a tumour-specific tracer.

In the E. coli and A549 cell culture assay, the results indicated that ^18^F-FLT uptake by *E. coli* was five times higher compared with that by tumour cells (35,680 and 7,200, respectively; P<0.01). This result indicates that bacteria in a reproductive stage take up a significantly higher amount of ^18^F-FLT compared with that taken up by tumour cells. In the rabbit *S. aureus* abscess model in the present study, the animals underwent ^18^F-FLT PET imaging after four weeks. The lesions were then removed for gross observation and histological examination. Samples were compared with lesions due to calcium sulphate + gentamicin to investigate the difference in the ^18^F-FLT uptake between the abscess and granuloma. After 40 days, the pathological changes in the right and left tibiae varied. In the side injected with *S. aureus*, a large number of inflammatory cells aggregated in the medullary cavity and abscess formation, necrotic bone formation and partial chronic osteomyelitis were also observed. These observations indicate that inflammatory changes and tissue repair were occurring simultaneously. In addition, a small degree of new bone formation was observed, replacing the granulation of fibrous tissue. In the side that received calcium sulphate and antibiotic, calcium sulphate was visible and the degradation products were surrounded by fibrous tissue. No inflammatory cells, local abscesses or bone necrosis were observed in the medullary cavity. Bilateral PET imaging results demonstrated that ^18^F-FLT uptake in the *S. aureus* abscess side was significantly higher compared with the calcium sulphate side (SUV_max_, 5.76±0.25 and 1.15±0.32, respectively; P<0.01).

^18^F-FLT is an indicator of cell proliferation, which is not unique to tumours. ^18^F-FLT uptake by inflammatory lesions depends on the growth period of the bacteria implanted in the animal model (19). During the bacterial reproductive period, the inflammatory bacteria exhibit exponential growth, accelerated DNA synthesis and increased ^18^F-FLT uptake. However, during the bacterial nonreproductive period or chronic period of bacterial inflammation, no bacterial reproduction occurs. Thus, ^18^F-FLT uptake is low. In addition, due to the immune reaction or formation of granulomas in the lesion, the tracer uptake in certain bacterial and nonbacterial infections during the development of the disease varies with the disease progression stage. Pathological changes differ at various stages (12). In the early stages, tuberculosis or other types of bacteria may multiply and DNA synthesis may accelerate. By contrast, endogenous lymphocyte and macrophage proliferation may form granulomas. In addition, nodular regions in the lesions at the edge of the calcium sulphate side exhibit higher ^18^F-FLT uptake. Thus, the calcium sulphate side may be the site that forms granulomas. In the chronic phase, the number of granulomas does not increase and the uptake of macrophages from the peripheral blood results in the formation of hard granulomas. During this period, granulomas are mainly formed when macrophages stop proliferating and bacteria stop reproducing. As a result, ^18^F-FLT uptake by the bacteria may stop. Therefore, granulomatous lesions with different causes may exhibit various ^18^F-FLT uptake rates, which should be distinct according to the different disease stages. All these results indicate that ^18^F-FLT is not a specific tumour tracer, since it is also absorbed by inflammatory lesions associated with reproductive-stage bacteria or viruses. However, the uptake may significantly decrease during the chronic phase.

^18^F-FLT as an imaging agent has an advantage over ^18^F-FDG in the diagnosis of tumour cell proliferation and inflammatory lesions. This is due to the different levels of ^18^F-FDG uptake by inflammatory lesions at various disease stages. At the acute stage, the number of granulocytes, monocytes and macrophages may increase. These cells are metabolically active and consume large amounts of glucose via the hexose monophosphate pathway during chemotaxis and phagocytosis (20). As a result, ^18^F-FDG uptake may increase. In the chronic phase, Sugawara *et al* (20) hypothesised that macrophages and neutrophils also take up ^18^F-FDG. In the chronic granuloma phase, capillary distribution in the tissue is sparse. In hypoxic or anaerobic conditions, macrophages can adapt to the hypoxic environment through anaerobic glycolysis, which results in increased ^18^F-FDG uptake.

Notably, the Baicalin content in rabbit serum specimens is 9–16 times higher compared with human sera (21). Large amounts of endogenous thymidine compete with ^18^F FLT for the adenosine transporter and the active site of TK. This competition results in low ^18^F-FLT uptake in the rabbit model (21). However, whether the experimental results of the present study may be applied to clinical practice requires further investigation.
